# Parishin B blocking TRIB3-AKT1 interaction inhibits breast cancer lung metastasis

**DOI:** 10.3389/fphar.2024.1517708

**Published:** 2025-01-15

**Authors:** Xiongtao Cheng, Jianguo Sun, Shouhong Chen, Nan Wang, Weijing Tang, Zijian Xia, Yuhong Shu, Shouhong Gao, Zhipeng Wang, Xinxia Wang, Rongzi Shao, Jianxiong Cao

**Affiliations:** ^1^ Graduate School, Hunan University of Chinese Medicine, Changsha, Hunan, China; ^2^ Department of Oncology, The First Hospital of Hunan University of Chinese Medicine, Changsha, Hunan, China; ^3^ Department of Pharmacy, Second Affiliated Hospital of Naval Medical University, Shanghai, China; ^4^ Department of Oncology, Guangzhou Concord Cancer Center, Guangzhou, Guangdong, China; ^5^ Department of Pharmacy, Shanghai Jiahui International Hospital, Shanghai, China; ^6^ Department of Pharmacy, The 960th Hospital of PLA Joint Logistics Support Force, Jinan, China

**Keywords:** TRIB3-AKT1 interaction, breast cancer lung metastasis, Parishin B, Co-IP assay, RNA-seq

## Abstract

**Background:**

TRIB3 has been reported to mediate breast cancer (BC) proliferation and metastasis by interacting with AKT1, and blocking the interaction between TRIB3 and AKT1 can inhibit the progression of BC. Besides, inhibiting TRIB3 to turn “cold tumor” hot has also been proved to be an effective therapeutic strategy for BC. Thus, this study aim to find drugs that can bind to TRIB3 to inhibit BC progression, and further elucidate its mechanism.

**Methods:**

The possible inhibitors of TRIB3 were screened by high-throughput molecular docking, CETSA, and CO-IP assay. Then, the effect of TRIB3 inhibitor anti BC was assessed by CCK-8 assay, flow cytometry, plate colony formation assay, and transwell assay; and the RNA-seq was empolyed to study the potential mechanism of Parishin B (PB) anti-BC. Finally, the effect of TRIB3 inhibitor on BC lung metastasis *in vivo* was evaluated.

**Results:**

PB was screened as a possible inhibitor of TRIB3, and CETSA and CO-IP assay indicated that PB could target TRIB3 and block TRIB3-AKT1 interaction. In addition, PB exhibited good anti-BC activity without drug toxicity in normal breast cells by experiments *in vitro*, and RNA-seq analysis suggested PB could inhibit the proliferation and invasion of BC cells related with cell cycle. It was also proved that PB could inhibit BC lung metastasis *in vivo*.

**Conclusion:**

The study demonstrated PB can bind to TRIB3 to inhibit BC proliferation and lung metastasis by blocking TRIB3-AKT1 interaction and regulating cell cycle, providing a therapeutic agent for the treatment of BC.

## 1 Introduction

According to the Global Cancer Observatory, breast cancer (BC) is the second most prevalent cancer in the world after lung cancer as of 2022 (Bray et al., 2024). Currently, the main treatment methods for breast cancer include surgery, radiotherapy, chemotherapy, and immunotherapy (Katsura et al., 2022; Kundu et al., 2024). Despite the constant improvement of treatment strategies and disease control for BC patients, metastasis, postoperative recurrence, immune escape, and chemotherapy resistance have led to unsatisfactory survival and prognosis for BC patients (Green, 2022; Raheem et al., 2023; Trapani et al., 2022). BC metastasis is the main cause of its postoperative recurrence and death (Yofe et al., 2023). A large number of studies have confirmed that the most common target organ for BC metastasis is the lung, so inhibiting lung metastasis of breast cancer is an important therapeutic strategy for the treatment of BC (Ibragimova et al., 2023). The emergence of immune checkpoint inhibitors has significantly altered the therapeutic outcomes of BC, but the differences in the tumor microenvironment between cold and hot tumors have resulted in very low remission rates and survival in BC patients with cold tumors (Cho and Kim, 2024). Cold tumors have fewer immune cell infiltrations in their tumor microenvironment and relatively low immune activity, making it difficult for immunosuppressive agents to be effective and resulting in poor immunotherapy outcomes. In contrast, hot tumors have a significant immune cell infiltration in their tumor microenvironment, such as high enrichment of effector T cells, making them more sensitive to treatment with immunosuppressive agents (Bonaventura et al., 2019). Targeted inhibition of TRIB3 has been shown to turn “cold tumor” hot and play an important role in BC progression (Shang et al., 2022).

Tribbles homolog 3 (TRIB3) is a member of the pseudokinase family, many studies have shown that TRIB3 is highly expressed in breast cancer, which is closely related to tumor progression and poor prognosis (Hu et al., 2024). The structure of TRIB3 protein contains three structural domains: the central kinase domain, the N-terminal domain, and the C-terminal domain; of which the central kinase domain lacks kinase activity due to deficiency ATP-binding sites, whereas the N-terminal domain is mainly associated with the binding of transcription factors (Orea-Soufi et al., 2021). Notably, TRIB3 is key stress regulators in a variety of tumors because its C-terminal domain can interact with ubiquitin ligases and various other proteins to promote tumor progression, such as AKT1, β-catenin, SQSTM1, TRIM8, EGFR, and TCF4 (Xiao et al., 2024; Yu et al., 2019; Zhang et al., 2020). Yu et al. showed that the C-terminal structural domain of TRIB3 in breast cancer interacts with AKT1 to interfere with FOXO1-AKT1 interactions and inhibit FOXO1 phosphorylation and ubiquitination, which further promotes the expression of the transcription factor SOX2 and confers stemness on BC cells to promote their proliferation and migration (Yu et al., 2019). These results suggest that targeting TRIB3 to block the TRIB3-AKT1 interaction is an effective therapeutic strategy for BC. Currently, TRIB3 inhibitors based on peptide strategies are still in the laboratory stage and there are no small molecule inhibitors of TRIB3 available. Therefore, there is a need to develop small molecule inhibitors of TRIB3 for BC therapy.

**FIGURE 1:**
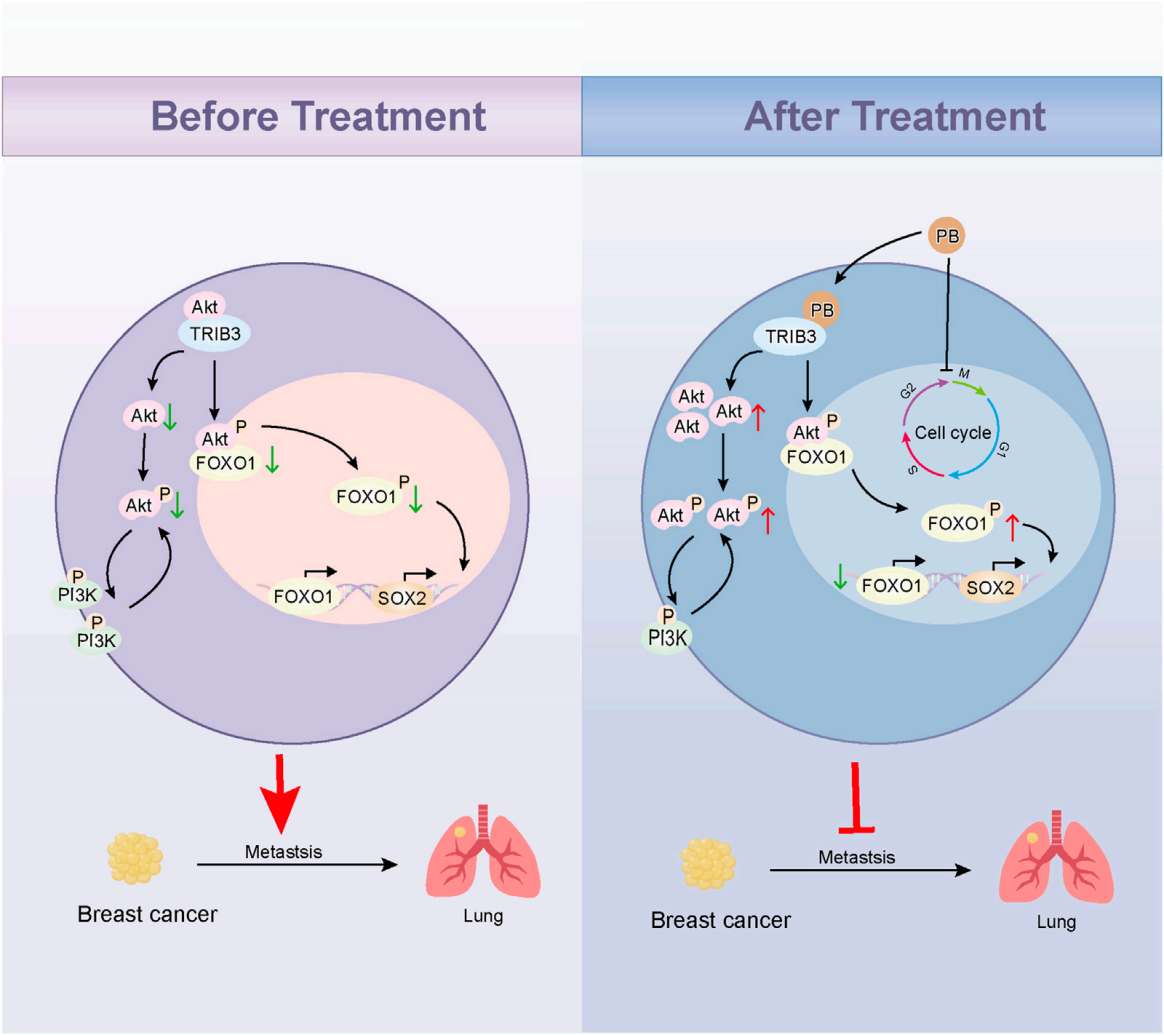
Graphical abstract.

Here, we determined that Parishin B (PB) could target and inhibit TRIB3 by high-throughput molecular docking screening combined with CETSA. CO-IP assay results showed that PB could block the binding of TRIB3 to AKT1; *in vitro* experiments confirmed that PB could inhibit the proliferation and migration of BC cells, and *in vivo* further confirmed that PB could inhibit the lung metastasis of BC. We then revealed the key mechanism of PB inhibiting BC progression by transcriptomics and validated it *in vitro* and *in vivo*. Our study developed a novel small molecule antagonist of TRIB3 and emphasized the therapeutic potential of PB for BC (Figure 1).

## 2 Materials and methods

### 2.1 Chemicals and regents

The standards of Parishin B (PB, CAS: 174972-79-3) (Lot: MUST-240711) (purity >98%) was provided by Chengdu MUST Co., Ltd. (Chengdu, China). The Cell Counting Kit-8 (CCK-8) (Batch No. ML-24082133), DMEM/F12 medium, Leibovitz’s L-15 medium, and phenylmethanesulfonyl fluoride (PMSF) were purchased from Meilun Biotech (Dalian, China). Fetal bovine serum (FBS) was purchased from Gibco (Australia). The Annexin V-FITC/PI Apoptosis Detection Kit and Cell Cycle and Apoptosis Analysis Kit were purchased from MedChemExpress Biotech (Shanghai, China). PVDF membranes (0.25 μm) were sourced from Millipore (Bedford, Massachusetts). Furthermore, RIPA lysis buffer, BCA assay kit, 12% SDS-PAGE gel, and Fast Blocking Western reagent were obtained from Beyotime (Shanghai, China). The TRIB3 antibody (batch no. ab75846) was procured from Abcam Biological Co., Ltd. (Shanghai, China). The AKT1 antibody (batch no. sc-5298) and Phospho-AKT1^Ser473^ antibody (batch no. sc-293125) were purchased from Santa Cruz Biotechnology. The Phospho-PI3K^Tyr458^ antibody (#17366) and the PI3K antibody (#4349) were procured from Cell Signaling Technology Co., Ltd. (Massachusetts, United States). In addition, the GAPDH antibody (60004-1-Ig), CDK1 antibody (67575-1-Ig), Cyclin B1 antibody (67686-1-Ig), and HRP-conjugated Affinipure secondary antibody (#SA00001-2) were provided by Proteintech Co., Ltd. (Wuhan, China).

### 2.2 Cell culture

Human BC cell lines MDA-MB-231, MDA-MB-231-LUC, SK-BR-3, MCF-7, HCC1954, T-47D, and the normal human mammary epithelial cell line MCF-10A were procured from the Cell Bank of the Chinese Academy of Sciences. All cell lines have been authenticated using short tandem repeats (STR) profiling within the last 3 years. All experiments were performed with mycoplasma-free cells. MDA-MB-231 was cultured in Leibovitz’s L-15 medium, while DMEM/F12 medium was used for culturing MCF-10A cells. All media were supplemented with 10% FBS, 100 U/mL penicillin, and 100 μg/mL streptomycin. The cells were maintained at 37°C in an incubator with an atmosphere containing 5% CO_2_. Cells were passaged until reaching 80% confluency and then transferred to 10 cm dishes for further experiments.

### 2.3 Molecular docking and molecular dynamic simulation

Referring to previous studies (Yu et al., 2019), retrieve the amino acid sequence of TRIB3 (NP_066981.2) from NCBI, and use Discovery Studio 2019 (BIOVIA) for protein homology modeling to obtain the protein structure of TRIB3, and the TRIB3 was chosen as receptors. Nearly 50,000 compound structures from PubChem database (https://pubchem.ncbi.nlm.nih.gov/) were selected as ligands. Subsequently, high-throughput molecular docking was employed to screen the potential inhibitors of TRIB3. Referring to previous studies (Liao et al., 2023), PyMol was used for TRIB3 protein preprocessing, including removing water molecules, adding polar hydrogens, and assigning Kollman charges. The docking box were determined using CASTp 3.0 web server (Tian et al., 2018), and molecular docking were performed with AutoDock 1.5.7 software. The TRIB3 inhibitors were selected through ranking the binding energy, and PyMol was applied to visualize docking conformation. Moreover, molecular dynamics simulation was used to assess the binding of inhibitors to TRIB3. Before starting the simulation, force field parameters for inhibitors were generated using CGenFF (Karwounopoulos et al., 2023), which were then applied to all atomic force fields and simulated with DESMOND (Oanca et al., 2023). Steepest descent algorithm was used to optimize the energy of the solvation system, and followed by equilibration using NVT and NPT couplings. Besides, short-range electrostatics were controlled by Coulomb and Van der Waals forces with a time step of 30 femtoseconds (fs), while long-range electrostatics were maintained through ParticleMesh Ewald (PME). Finally, molecular dynamics simulations at 300K were run for 100 nanoseconds (ns), and results include root mean square deviation (RMSD), root mean square fluctuation (RMSF), and hydrogen bonds number bonding from the trajectories.

### 2.4 Cellular thermal shift assay (CETSA)

MDA-MB-231 cell lysate was obtained using RIPA lysis buffer. The MDA-MB-231 cell lysates were divided into two aliquots, which were treated with 10 μM PB and an equal volume of DMSO for 1 h, respectively. Each treatment group was then further divided into six equal portions, which were incubated at temperatures of 35, 40, 45, 50, 55, and 60°C for 10 min, followed by a 3-min cooling period. Subsequently, the heated lysates were centrifuged at 17,400 g for 15 min at 4°C. The resulting supernatants were then subjected to Western blot analysis. The melting curve was fitted using the Boltzmann sigmoidal function in GraphPad.

### 2.5 Co-immunoprecipitation (CO-IP)

The MDA-MB-231 cells were treated with 20 μM PB or DMSO for 24 h, and then collected. Cells were lysed using RIPA lysis buffer containing PMSF. Anti-TRIB3 antibody (batch no. ab75846) was used as the “bait” antibody to immunoprecipitate TRIB3, or alternatively, anti-AKT1 antibody (batch no. sc-5298) was used for immunoprecipitation. Protein A/G agarose beads (catalog number P2055, Beyotime) were employed to capture the immune complexes. The immunoprecipitates were thoroughly washed to remove non-specifically bound proteins. Western blot analysis was performed to detect the proteins in the immunoprecipitates. Anti-TRIB3 or anti-AKT1 antibodies were used to probe the corresponding proteins in the immunoprecipitates.

### 2.6 RNA sequencing and analysis

The MDA-MB-231cells in two groups (control and 20 μM PB treatment group) were collected for RNA-seq (n = 3 for each group). These cell samples were processed using CloudSeq mRNA enrichment kit and Illumina HiSeq sequencing (Thermo Fisher Scientific, MA, United States) by GENEWIZ Biotech Co., Ltd. (Suzhou, China), and the original data is stored in the GEO database (GEO ID: GSE285440). Differentially expressed genes (DEGs) between the groups were identified using the DESeq2 Package. The thresholds of the DEGs were set as log |Fold-change| ≥ 2 and *P* ≤ 0.05 with fragments per kilobase million (FPKM) value ≥ 0.1. Thereafter, the protein-protein interaction (PPI) network of DEGs was constructed by STRING databases, and the degree of the network was calculated to carry out the key targets using Cytoscape software. Moreover, The GO and KEGG enrichment analysis were employed to further confirm DEGs functions using ClusterProfiler and org. Hs.e.g.,.db packages, and the analysis results were ranked and aggregated to find mechanism of PB against BC. Finally, terms with the highest scores were displayed by the ggplot2 package.

### 2.7 Flow cytometry

The apoptosis rate and cell cycle of the MDA-MB-231 cell line were assessed using flow cytometry, the concentrations of PB were set at 5 μM, 10 μM, and 20 μM, respectively. The MDA-MB-231 cells were seeded in 6-well plates at a density of 5*105 per well and cultured in an incubator for 24 h, followed by treatment with different concentrations of PB. After a 24-h treatment period, EDTA was added to each group to detach and collect the MDA-MB-231 cells, and cells were stained to analysis the apoptosis rate by the Annexin V-FITC/PI double staining assay. Besides, the effect of PB on the cell cycle was assessed using Cell Cycle Analysis Kit. Flow cytometry analysis was performed on each sample containing a minimum of 1*104 cells, and FlowJ software was used to analyze and display the results.

### 2.8 Colony formation assay

To assess cell proliferation, MDA-MB-231 cells (1*103 cells/well) were seeded in 6-well plates, and treated with PB at different concentrations. The concentrations of PB were set at 5μM, 10μM, and 20μM, respectively. The culture medium was replenished every 2 days during a 12-day period. Then, the cells were washed three times with PBS and fixed with paraformaldehyde for 30 min. Following the fixation, staining was performed using a 0.1% crystal violet solution for 20 min. Images were captured, and colony counts were quantified using ImageJ software.

### 2.9 Transwell invasion and migration assay

The migration and invasion effects of PB on MDA-MB-231 cells were detected by Transwell assays. First, MDA-MB-231 cells (7 × 104 cells in 200 µL serum-free L-15 medium) were seeded into the upper chamber of a 24-well plate, while the lower chamber was filled with 700 µL of corresponding medium containing 20% FBS. After incubation for 24 h, migrated cells were fixed with 4% paraformaldehyde for a duration of 30 min and subsequently stained with a solution of crystal violet at a concentration of 0.1% for a period of 20 min. Following the three washes with PBS, non-migrated cells were eliminated from the upper chamber using a cotton swab. Randomly selected migrated cells were visualized under a microscope and quantified utilizing ImageJ software. Different from the migration assay, the invasion assay is conducted in Transwell chambers precoated with Matrigel (at a concentration of 2 mg/mL). The Matrigel is incubated at 37°C for 30 min to form a solid layer. The assay is then performed following the same procedure as the migration assay, using Transwell inserts with a pore size of 8 µm.

### 2.10 Western blotting

The total protein was extracted from MDA-MB-231 cells or mouse tissue samples using RIPA Lysis buffer mixed with PMSF buffer at 4°C, and the protein concentrations was detected by BCA method. The proteins were then denatured at high temperature for 10 min, and loading buffer was used to prepare the total protein. Next, equivalent proteins samples were added to 12% SDS-PAGE gels to electrolyze and isolate at constant voltage (150 V), and then gels were transferred to PVDF membranes and sealed in 5% skim milk powder for 1 h. Primary antibodies were then incubated overnight at 4°C on the membranes. Following primary incubation, corresponding HRP-conjugated secondary antibodies were applied to the membranes and incubated at room temperature for 1 h. Finally, ECL A and B (mixed in a ratio of 1:1) were applied evenly to the membranes for signal detection through chemiluminescence imaging. Signal quantification was performed using ImageJ software, and GAPDH was used as an internal reference.

### 2.11 Overexpression TRIB3

The procedure for overexpressing TRIB3 in MDA-MB-231 cells is as follows: Clone the human TRIB3 cDNA into the pcDNA3.1-HA vector, then transfect MDA-MB-231 cells using Lipofectamine 3000 transfection reagent. After 24 h of transfection, replace the cell culture medium and continue to culture. Finally, verify the efficiency of overexpression using Western blot assay.

### 2.12 Animal experiments

Female BALB/c nude mice, aged 4–5 weeks, were obtained from Shanghai Yishang Biotechnology Co., Ltd [License number: SCXK-2022–0011] and were housed in the animal Experiment Center at Shanghai University of Traditional Chinese Medicine. All animal experiments were conducted with the approval of the Yishang Technology Co., Ltd Animal Ethics Committee and in compliance with ethical standards and national guidelines [License number: SCXK-2024-Mi-032].

To investigate the impact of PB on BC lung metastasis (BCLM) *in vivo*, mice were randomly divided into four groups: Control, PB low, PB high, and paclitaxel (PTX). The mice have unrestricted access to water and food. Each mouse was injected with MDA-MB-231-LUC cell suspensions (2 × 106 cells in 200 µL PBS) via the tail vein to prepare a BC mouse model. Subsequently, mice were treated with PB or PTX as follows: PB was administered via intraperitoneal injection once daily at dosages of 4 mg/kg and 8 mg/kg, respectively. PTX was administered via intraperitoneal injection twice a week at a dose of 4 mg/kg, based on previous research. After a duration of 6 weeks, the mice were euthanized, and the total number of metastatic tumor nodules in five lobes of the lung tissue was quantified. Additionally, the overall fluorescence intensity of the lungs was measured using an *in vivo* imaging system. Afterward, the lung tissues were collected for subsequent experiments.

### 2.13 Hematoxylin and Eosin (H&E) staining

H&E staining was employed to evaluate the pathological features of mouse lung tissue. Briefly, After the final administration, lung tissues from the mice were collected, followed by paraffin embedding and sectioning. The sections were deparaffinized using xylene and rehydrated through a graded alcohol series. Hematoxylin was used to stain nuclei, followed by a quick acid-alcohol rinse for differentiation. Eosin was applied to stain cytoplasmic structures. After dehydration through increasing alcohol concentrations and clearing with xylene, the slides were prepared for microscopic examination.

### 2.14 Statistical analysis

Statistical analysis and visualization were conducted using GraphPad Prism 9.0, with results presented as mean ± SEM. Comparisons between groups were performed using the Student’s t-test and one-way analysis of variance (ANOVA). P < 0.05 (*), P < 0.01 (**) and P < 0.001 (***) were considered statistically significant.

## 3 Results

### 3.1 PB can target and inhibit TRIB3

**FIGURE 2:**
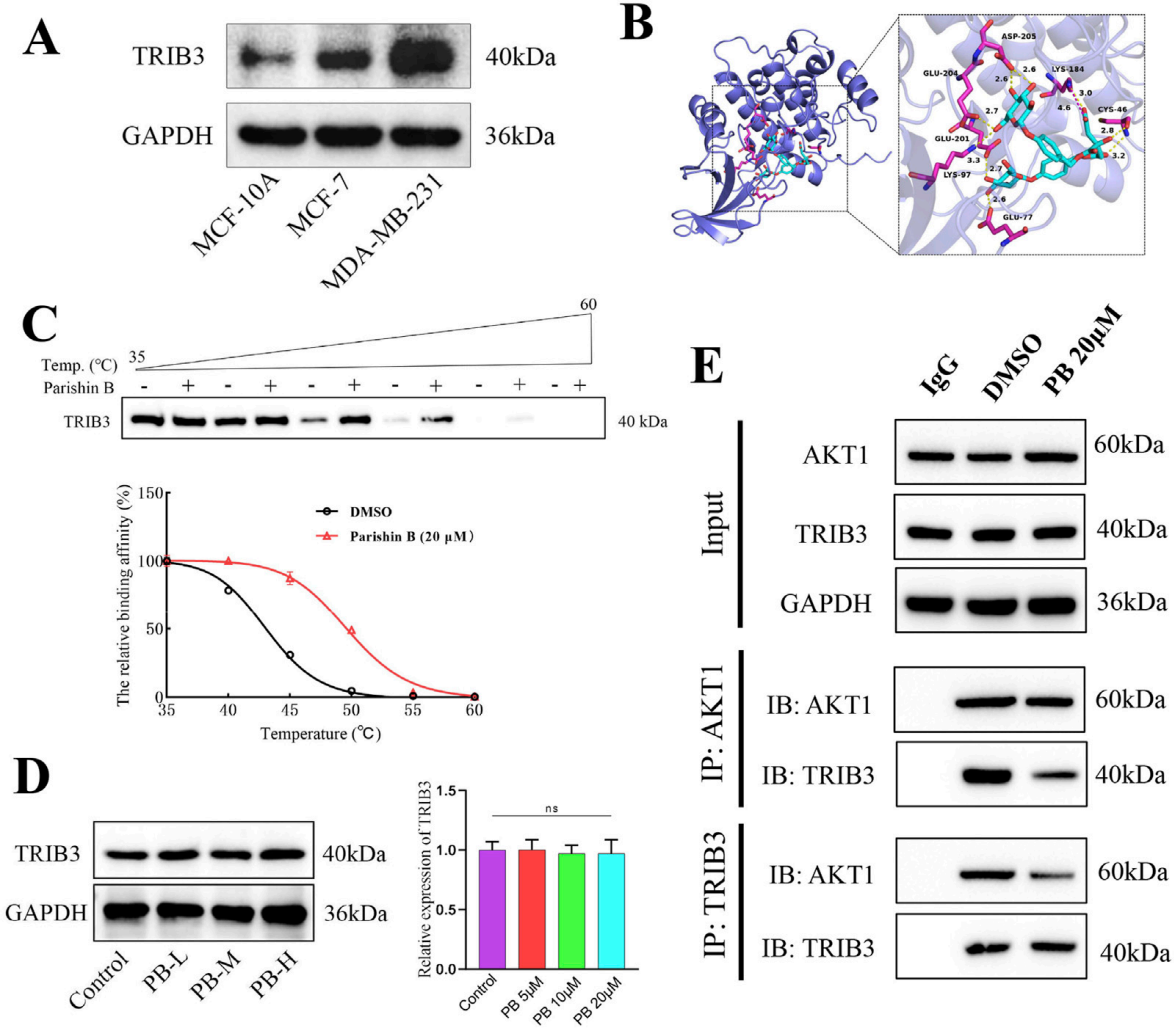
PB binding TRIB3 and blocking TRIB3-AKT1 interaction. **(A)** TRIB3 is highly expressed in Breast Cancer Cells; **(B)** Molecular docking screening for molecules that can bind to TRIB3; **(C)** CETSA assay to assess the bind effect of PB to TRIB3; **(D)** The effect of PB on the expression of TRIB3 in MDA-MB-231 cells; **(E)** Co-IP assay to detect PB binding TRIB3 and blocking TRIB3-AKT1 interaction.

Given that TRIB3 is highly expressed in BC tissues (Figure 2A), supports cancer stemness, and promotes tumor progression, inhibiting TRIB3 is suggested as a promising therapeutic strategy for BC (Lee et al., 2019; Zhang et al., 2024). We initially discovered that PB binds tightly to TRIB3 through high-throughput molecular docking screening, suggesting it may serve as a potential TRIB3 inhibitor (Figure 2B; Supplementary Table S1). Subsequently, we conducted 100 ns molecular dynamics simulations of PB and TRIB3. The results indicated that the final RMSD of the ligand stabilized at approximately 6.0 Å after fluctuation, suggesting that PB and TRIB3 form a more stable binding conformation compared to the initial TRIB3 structure (Supplementary Figure S1A). The RMSF values for PB-TRIB3 binding were generally acceptable (Supplementary Figure S1B). Furthermore, the results identified ASP205 and GLTJ201 as the primary amino acid residues involved in PB’s binding to TRIB3, playing a crucial role in this interaction (Supplementary Figure S1C-E). We also validated the targeted inhibition of TRIB3 by PB using a CETSA assay, which demonstrated that PB enhances the thermal stability of TRIB3, resulting in improved thermal stabilization of TRIB3 and a rightward shift in the melting curve (Figure 2C). Then, we assessed the effect of PB on TRIB3 protein levels in the BC cell line MDA-MB-231 using Western blotting. The results indicated that PB no significantly effect on TRIB3 expression in MDA-MB-231 cells (Figure 2D). Additionally, results of CO-IP assay indicate that PB can bind with TRIB3 to blocking the TRIB3-AKT1 interaction (Figure 2E). In summary, PB can targeting TRIB3 and blocking the TRIB3-AKT1 interaction, positioning it as a novel potential TRIB3 inhibitor and therapeutic agent for BC.

### 3.2 PB inhibits the proliferation, invasion, and migration of BC cell lines *in vitro*

**FIGURE 3:**
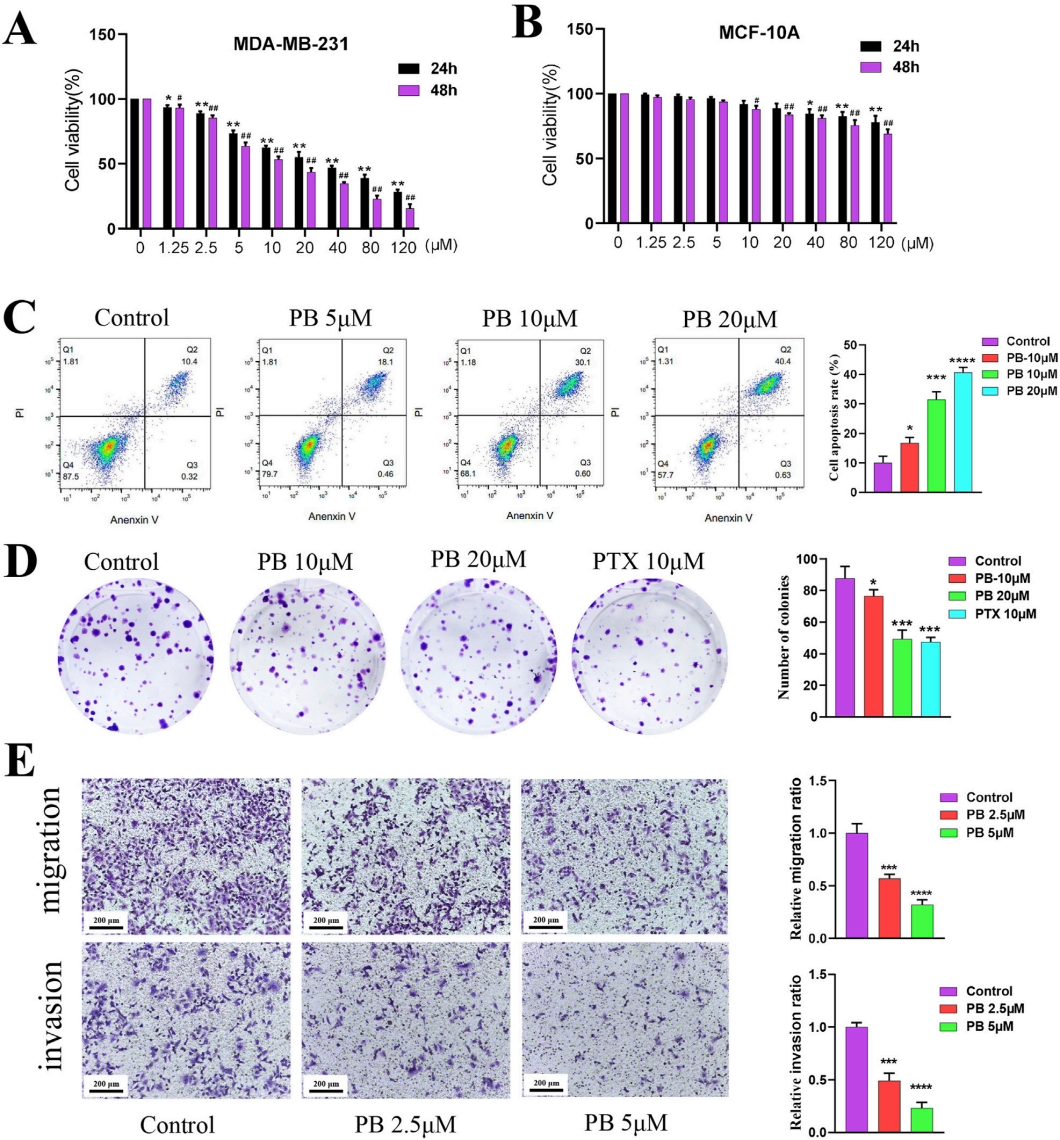
The effect of PB anti-BC *in vitro*. **(A, B)** CCK-8 assay was employed to evaluate the proliferation of BC cells (MDA-MB-231) and normal breast epithelial cells (MCF-10A) treatment with PB; **(C)** Flow cytometry was used to detect the effect of PB on BC cells apoptosis; **(D)** The effect of PB on the cloning ability of BC cells was detected by colony-formation assay; **(E)** The Transwell assay was uesd to assess the invasion and migration abilities of BC cells treatment with PB.

To evaluate the potential impact of PB on the progression of BC, we first evaluated its proliferative toxicity on five types of BC cells using a CCK8 assay, including MDA-MB-231, SK-BR-3, MCF-7, HCC 1954, and T-47D, the results indicated that PB had the best effects on MDA-MB-231 and MCF-7 cells (Figure 3A; Supplementary Figure S2A-D). Then, transwell assay was employed to assess the impact of PB on the migration of these two types of BC cells, and results demonstrated that PB had the most pronounced inhibitory effect on the migration of MDA-MB-231 cells (Supplementary Figure S2E). In addition, we also assessed the proliferative toxicity of PB on normal mammary cells, and results revealed that PB without exhibiting notable toxicity to normal mammary cells, indicating a selective cytotoxic effect on cancer cells (Figure 3B). Based on the results, we subsequently selected MDA-MB-231 as the subject of our study. Considering that 10 μM of PB exhibited cytotoxicity to MCF-10 cells, the concentrations of PB were set at 5, 10, and 20 μM. Then, result of flow cytometry indicate that PB can induceBC cells apoptosis (Figure 3C); besides, colony formation assays confirmed that PB markedly suppressed the proliferation of BC cell lines (Figure 3D). Furthermore, Transwell invasion and migration assays were conducted to examine the effects of PB on the invasion and migration of BC cells (Figure 3E). The results demonstrated that PB significantly reduced both invasive and migratory capabilities of MDA-MB-231 cells. In summary, these findings underscore the potent anti-breast cancer effects of PB.

### 3.3 The mechanisms of PB against breast cancer analysis by RNA-seq

**FIGURE 4:**
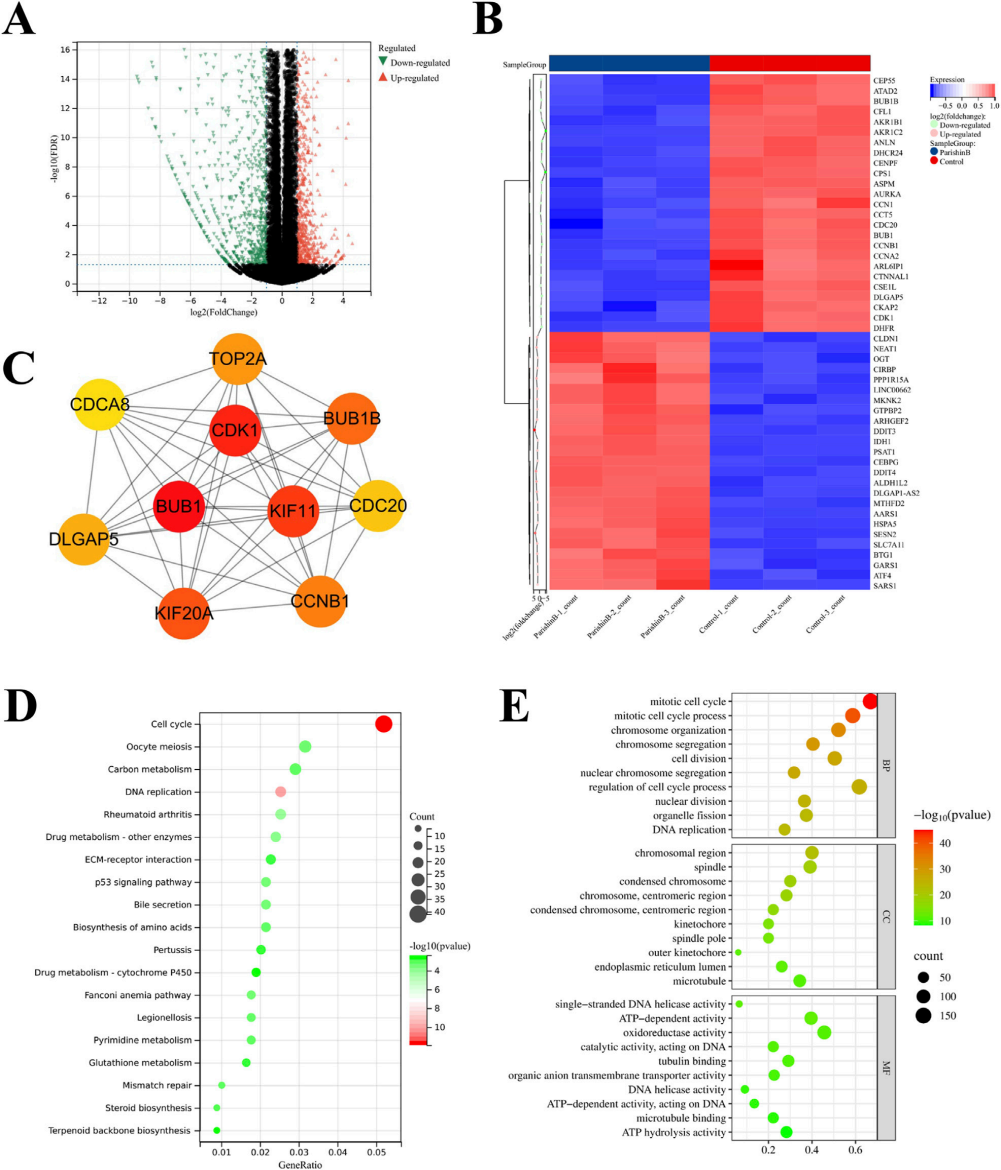
Transcriptome analysis of BC cells treatment with PB. **(A)** Differentially expressed genes (DEGs) screening; **(B, C)** The core targets of PB anti-BC identified; **(D)** KEGG enrichment analysis of DEGs; **(E)** GO enrichment analysis of DEGs.

To further investigate the targets and mechanisms underlying PB’s anti-BC effect, RNA-seq was conducted on samples from the control and PB-treated groups (20 μM), and differentially expressed genes (DEGs) after 24 h of treatment were identified using the DEseq2 program. Volcano plot analysis revealed a clear distinction between the two groups, indicating that PB could affect the gene expression profile of MDA-MB-231 cells (Figure 4A). Additionally, heatmap and PPI network analysis identified genes with significant expression changes following PB treatment, such as CDK1, BUB1, and KIF11 and others (Figures 4B, C). KEGG pathway enrichment analysis of DEGs highlighted the top 20 signaling pathways were most associated with PB treatment, with cell cycle signaling pathways playing a crucial role in PB’s anti-breast cancer effects (Figure 4D). Next, the GO enrichment analysis was performed and the results showed that the DEGs were mainly enriched in mitotic cell cycle, mitotic cell cycle process, and chromosomal region, etc. (Figure 4E). In conclusion, PB can targeting TRIB3 and disrupting the cell cycle signaling pathway in BC, offering deeper insights into the mechanisms underlying PB’s anti-cancer effects.

### 3.4 PB blocks the G2/M phase of the cell cycle to exert anti-BC effects

**FIGURE 5:**
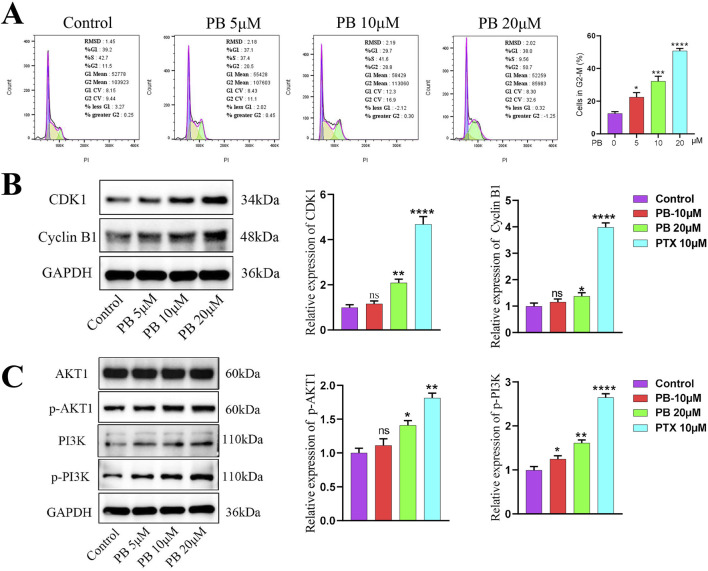
PB can regulates cell cycle and PI3K/Akt signaling pathway. **(A)** Flow cytometry was employed to evaluate the cell cycle changes of BC cells treatment with PB; **(B)** The expression level of CDK1 and Cyclin B1 in BC cells after PB treat; **(C)** PB promotes the phosphorylation levels of AKT and PI3K in BC cells.

KEGG enrichment analysis identified the cell cycle as key mechanisms through which PB acts against BC. Blocking the tumor cell cycle is a well-established anti-cancer strategy. We first examined the cell cycle of MDA-MB-231 cells following PB treatment using flow cytometry, which showed that PB significantly inhibited the G2/M phase, leading to reduced cell proliferation (Figure 5A). Additionally, Western blot assay was used to measure the expression level of cell cycle-related control proteins, including CDK1 and Cyclin B1, yielding results consistent with the flow cytometry findings (Figure 5B). These results demonstrate that PB effectively blocks the G2/M phase in BC cells, thereby inhibiting their proliferation.

The PI3K/Akt signaling pathway primarily functions through phosphorylation activation of PI3K and AKT, with AKT1 shown to interact with TRIB3, inhibiting FOXO1 degradation and promoting SOX2 transcription to enhance BC cell stemness (Yu et al., 2019). To explore the key pathway by which PB targets TRIB3 in breast cancer, we assessed the phosphorylation levels of PI3K and AKT in MDA-MB-231 cells after PB treatment. The results indicated a significant promote PI3K and AKT phosphorylation compared to the control group (Figure 5C). Then we verified the overexpression efficiency of TRIB3 through Western blot (Supplementary Figure S3A), and the subsequent results indicated that overexpressing TRIB3 could significantly reverse the effects of PB (Supplementary Figure S3B).

### 3.5 PB suppresses MDA-MB-231 cells metastatic colonization of the lung *in vivo*

**FIGURE 6:**
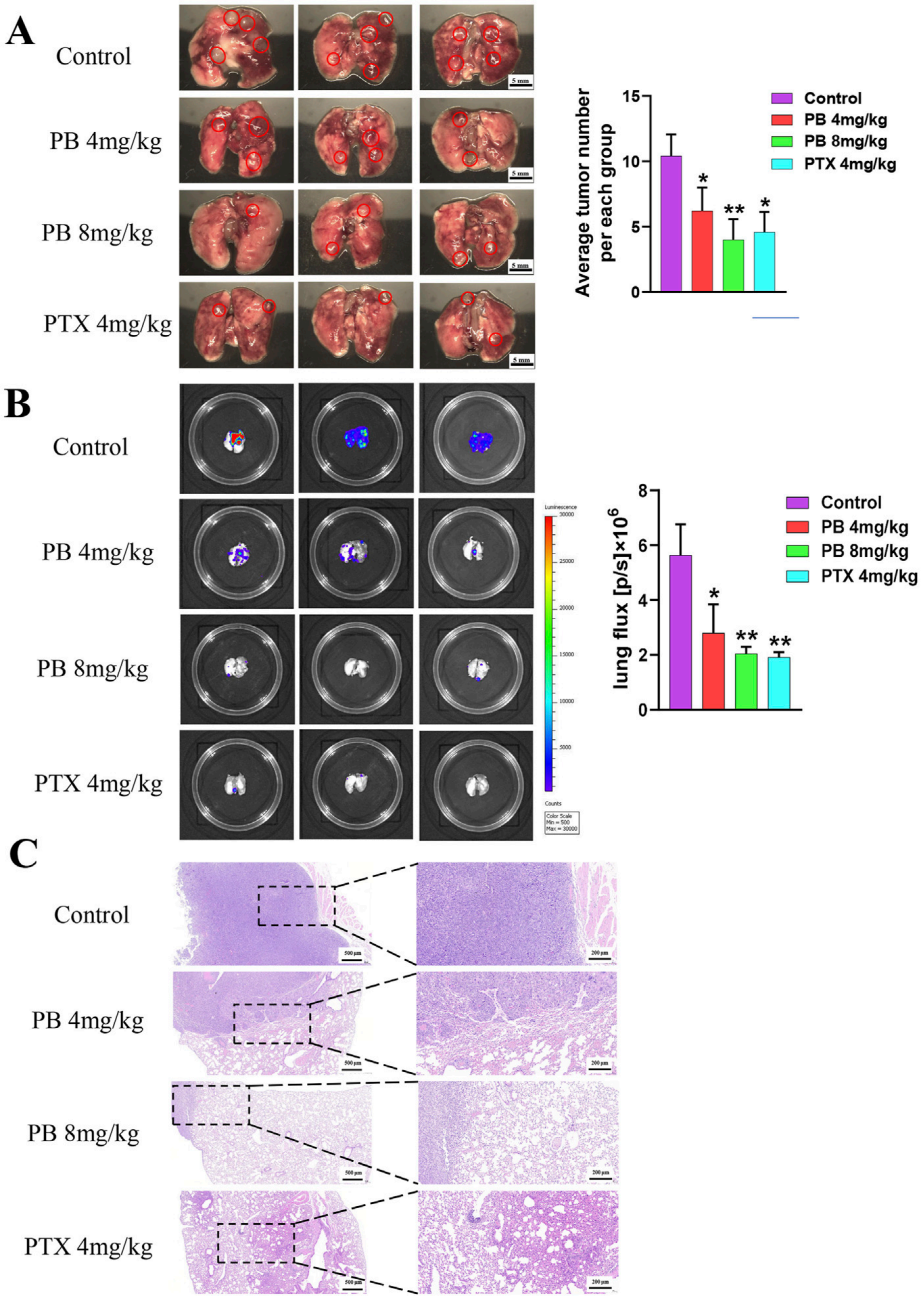
PB significantly inhibits lung metastasis of breast cancer in mice. **(A)** Lung metastatic tumor image; **(B)** fluorescence imaging; **(C)** H&E staining of lung tissue.

**FIGURE 7:**
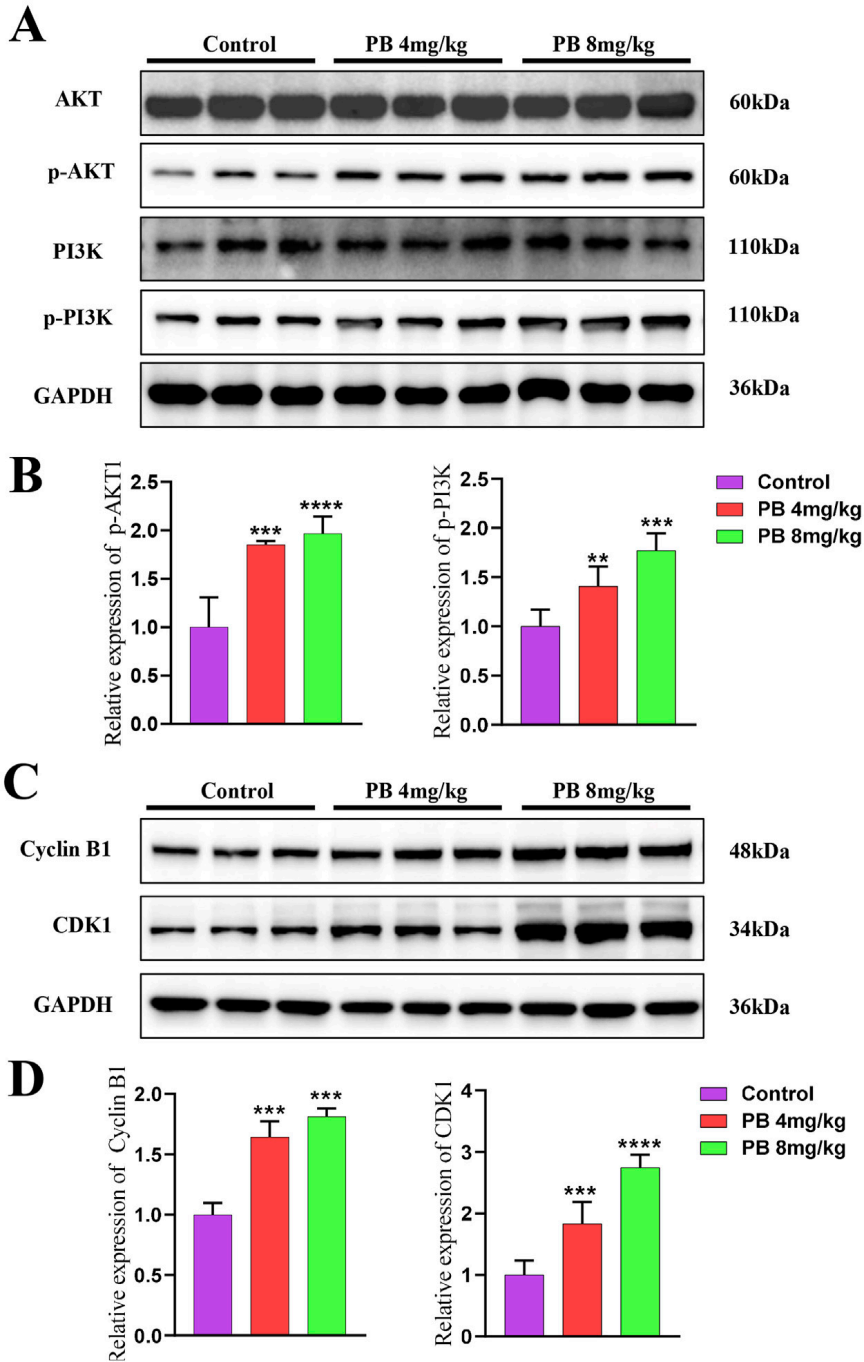
Mechanism validate *in vivo*. **(A, B)** PB promotes the phosphorylation levels of AKT and PI3K in mice; **(C, D)** PB upregulates CDK1 and Cyclin B1 expression, arresting cells in the G2-M phase of the cell cycle.

To further investigate PB’s role in inhibiting BC metastasis, MDA-MB-231-LUC cells were injected into nude mice via the tail vein to establish a lung metastasis model. PB or PTX was then administered intraperitoneally for four consecutive weeks. Our results showed a significant reduction in lung tumor nodules following treatment with PB or PTX (Figure 6A). Fluorescence intensity analysis of lung tissues confirmed a significant reduction in metastatic spread (Figure 6B). In addition, H&E staining showed marked improvement in pulmonary metastatic lesions in the PB-treated group compared to the control group (Figure 6C). These findings aligned with the *in vitro* results. Moreover, an evaluation of biochemical and organ indices after PB administration indicated no significant drug toxicity, with all organ indices remaining within normal ranges (Supplementary Figure S14). Then, We evaluated the level of CDK1 and Cyclin B1, The results indicate that PB significantly promotes the expression of CDK1 and Cyclin B1, thereby arresting the cell cycle at the G2-M phase (Figures 7A, B). Moreover, we also assess the phosphorylation levels of PI3K and AKT in the lung tissues of mice treated with PB, which yielded results consistent with those observed in the cell experiments (Figures 7C, D). Collectively, these results suggest that PB effectively inhibits the lung metastasis of BC cells and is a safe and promising therapeutic agent for BC.

## 4 Discussion

Despite significant advances in interventions such as surgery, radiotherapy, and chemotherapy for BC, lung metastasis remains a key obstacle, leading to treatment failure and mortality in BC patients. BC treatment continues to face substantial challenges (Valente and Roesch, 2024). Numerous studies have identified TRIB3 as an important tumor marker and therapeutic target, promoting cancer progression by interacting with various target proteins across multiple tumor types. In lung cancer, TRIB3 has been reported to facilitate tumor progression by interacting with EGFR (Yu et al., 2020); Similarly, TRIB3 has been shown to enhance the stemness of cancer cells and drive the progression of colorectal cancer and glioma by interacting with β-catenin and TCF4 (Hua et al., 2019; Lu et al., 2020). Moreover, TRIB3 has been found to interact with STAT3, mediating angiogenesis and promoting tumor metastasis (Chen et al., 2022). In BC, TRIB3 interacts with AKT1, disrupting the FOXO1-AKT1 interaction, which inhibits FOXO1 degradation. This inhibition subsequently enhances the expression of the transcription factor SOX2, contributing to the stemness, proliferation, and migration of BC cells (Xie et al., 2024; Yu et al., 2019). Collectively, these findings highlight that TRIB3 predominantly interacts with other proteins through its C-terminal KD structural domain. Targeting this domain to block these protein interactions presents a promising strategy for tumor therapy.

Parishin B (PB) is a phenolic compound isolated from the traditional Chinese medicine Gastrodia elata Blume, recognized as one of its primary active ingredients (Kim et al., 2019). Currently, PB has been reported to inhibit the progression of hepatocellular carcinoma by suppressing NF-κB activation in CD4^+^ T cells (Shu et al., 2013). Studies on PB’s anti-tumor effects are limited, existing research primarily focuses on the isolation, identification, metabolic distribution, and *in vivo* pharmacokinetics of PB, which demonstrated favorable absorption, distribution, and pharmacokinetic parameters (Tang et al., 2014). Additionally, PB has been established as a safe pharmaceutical ingredient, indicating its druggability. In this study, we discovered that PB binds to the KD structural domain at the C-terminal end of TRIB3 through high-throughput molecular docking screening. We further confirmed that PB could target binding to TRIB3 to block the binding of TRIB3 to AKT1 by CETSA, molecular dynamics, and CO-IP. Transcriptomic analysis allowed us to elucidate the anti-breast cancer mechanism of PB, and we confirmed its inhibitory effects on the proliferation, invasion, and migration of BC cells *in vitro*. Furthermore, *in vivo* experiments revealed that PB can inhibit lung metastatic colonization of BC. This study is the first to report that PB targets TRIB3 to disrupt the TRIB3-AKT1 interaction, thereby inhibiting lung metastasis in BC, highlighting its potential as a therapeutic agent for BC treatment.

Aberrant activation of the PI3K/Akt signaling pathway is known to significantly influence the proliferation, apoptosis, invasion, and migration of various tumor cells. The activity of this pathway is positively correlated with the activity of AKT kinase (Yuan et al., 2023). mTOR serves as a key substrate for the PI3K/Akt signaling pathway, and its S2448 site can be phosphorylated and activated by AKT, which subsequently regulates tumor cell energy metabolism and triggers processes such as autophagy and apoptosis (Glaviano et al., 2023). Notably, AKT also modulates the FoxO and NF-κB/Rel families of transcription factors, inhibiting FoxO transcription factor activity through phosphorylation to regulate downstream target genes, including SOX2 and TRADD (Schaefer and Lengerke, 2020). In the present study, we demonstrated that PB targets TRIB3 and disrupts the TRIB3-AKT1 interaction to inhibit BC metastasis. Interestingly, we did not find that PB could inhibit the activation of the PI3K/Akt signaling pathway; instead, blocking the TRIB3-AKT1 interaction led to an upregulation of AKT phosphorylation levels, which did not contribute to tumor progression. These findings align with previous studies indicating that peptide-mediated blockade of the TRIB3-AKT1 interaction also resulted in AKT activation without promoting BC progression (Yu et al., 2019). Additionally, other studies have shown that AKT activation does not always correlate with tumor advancement (Cheung and Testa, 2013). However, the precise mechanism by which AKT and upstream PI3K activation does not lead to tumor progression remains to be elucidated.

## 5 Conclusion

In conclusion, our study demonstrated that PB can target TRIB3 to block the TRIB3-AKT1 interaction, thereby inhibiting BC lung metastasis. However, there are several limitations to this study that warrant further exploration. Future research should focus on: 1) identifying other targets of PB that may inhibit BC progression; 2) elucidating the molecular mechanism by which PB blocks the interaction between TRIB3 and AKT1, as well as other proteins involved in BC progression; and 3) investigating potential negative physiological signals associated with PB’s activation of the PI3K/Akt pathway in BC inhibition.

## Data Availability

The data presented in the study are deposited in the GEO repository, accession number GSE285440.
