# Supplementary material for: Parishin B blocking TRIB3-AKT1 interaction inhibits breast cancer lung metastasis
Source: Front Pharmacol. 2025 Jan 15;15:1517708. doi: 10.3389/fphar.2024.1517708 (PMC11775015; doi:10.3389/fphar.2024.1517708)
Supplement: Supplementary file 1 [file DataSheet1.docx]

**Supplementary Figures:**


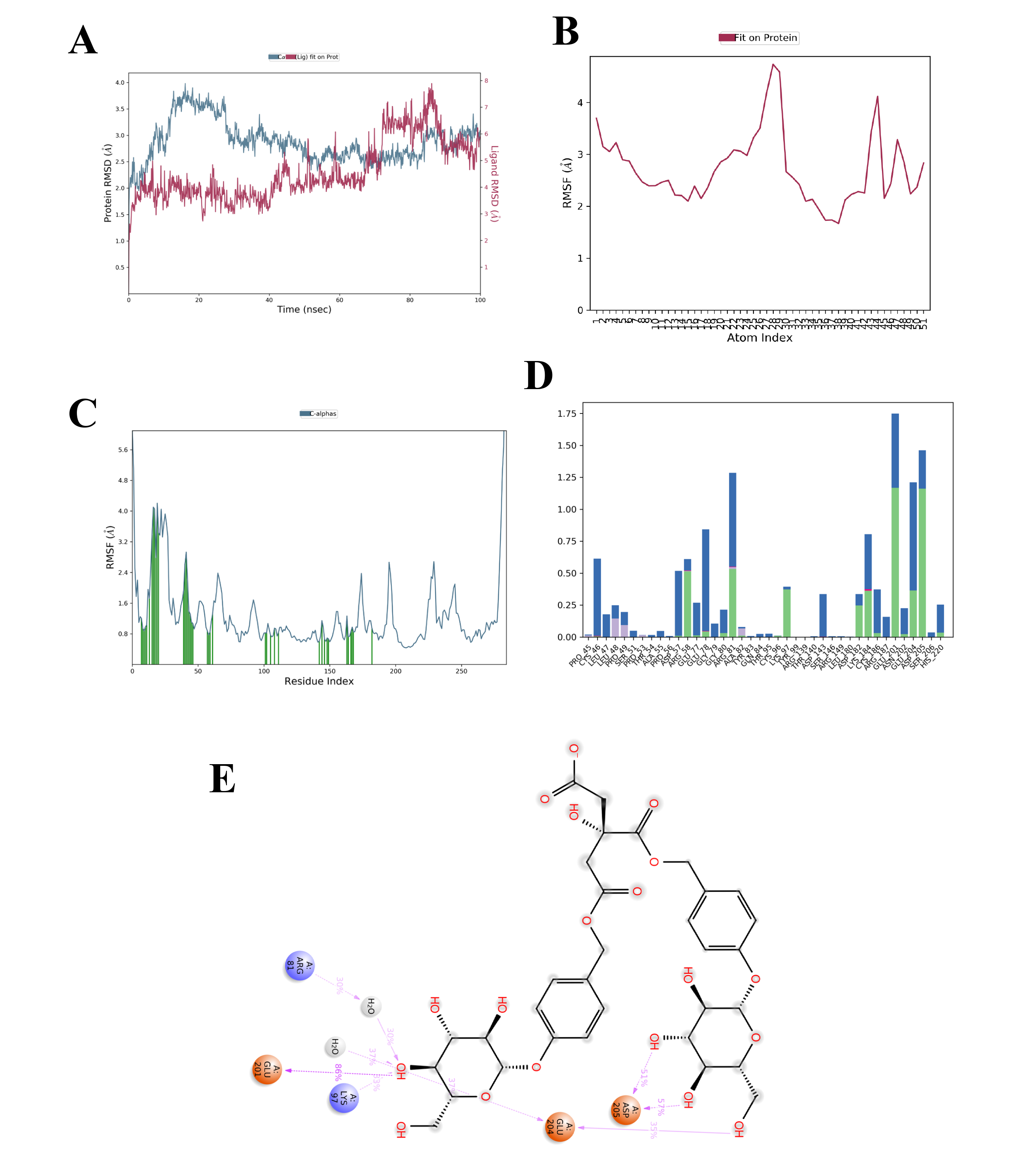
Fig.S1. Molecular dynamics simulation of PB binding to TRIB3. (A) RMSD value; (B-C) RMSF value; (D-E) The key binding sites of PB bind to TRIB3.

Fig.S2. The Impact of PB on some Breast Cancer Cells. (A) SK-BR-3; (B) MCF-7; (C) HCC1954; (D) T-47D; (E) The effect of PB on the migration of MCF-7 cells


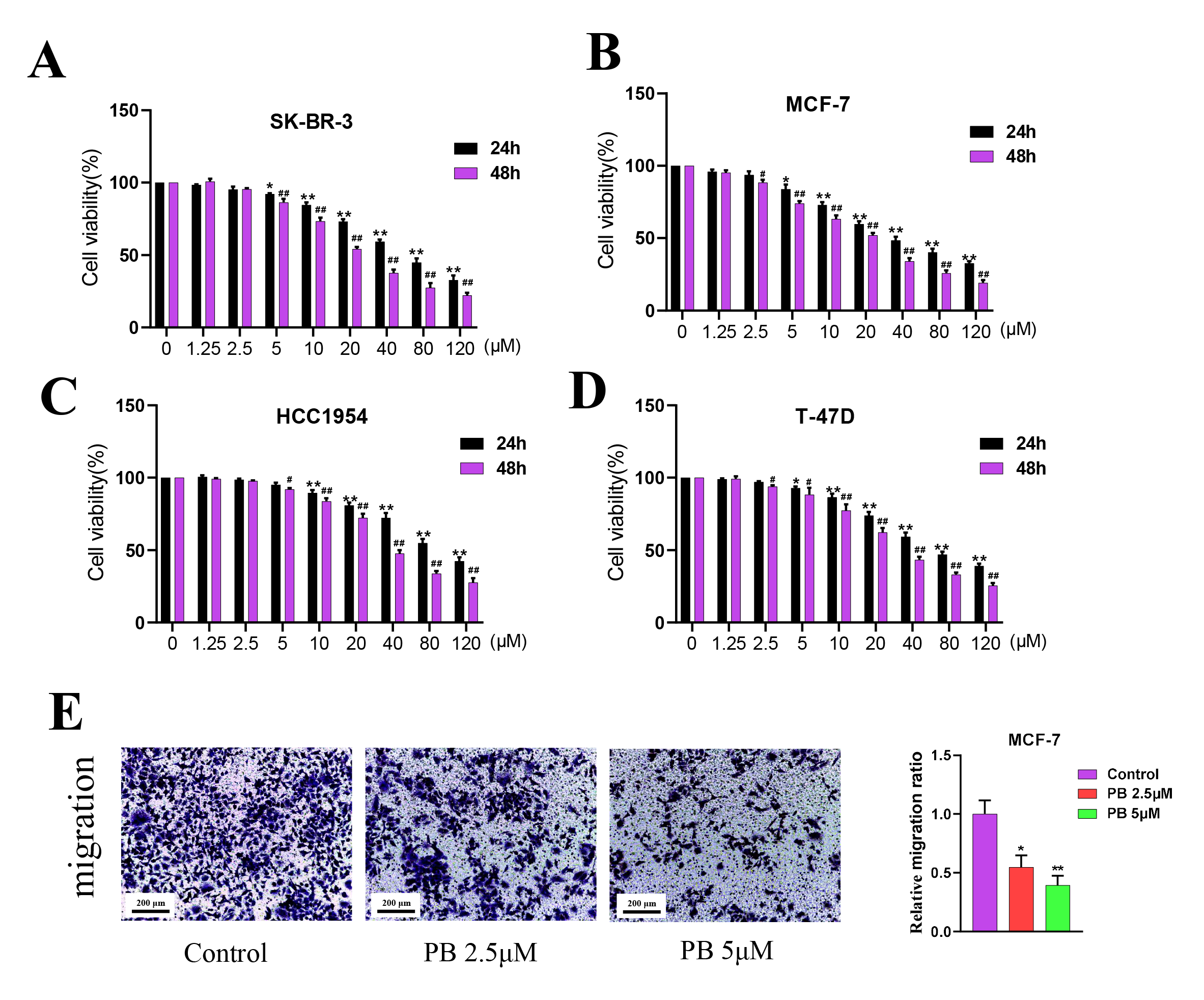


Fig.S3. Rescue experiment in vitro. (A) Validating TRIB3 overexpression; (B) Overexpression of TRIB3 can rescue the therapeutic effect of PB


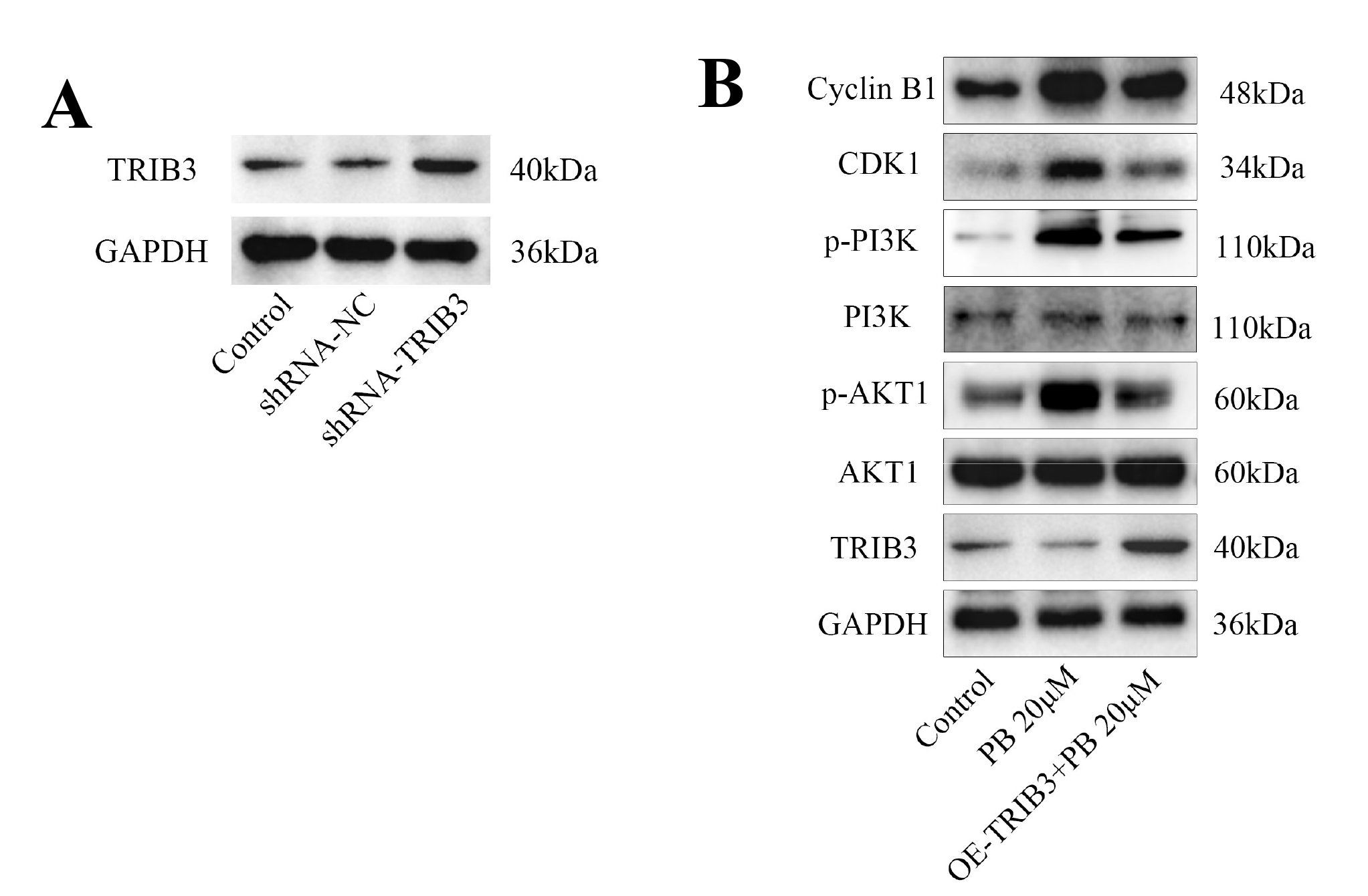


**
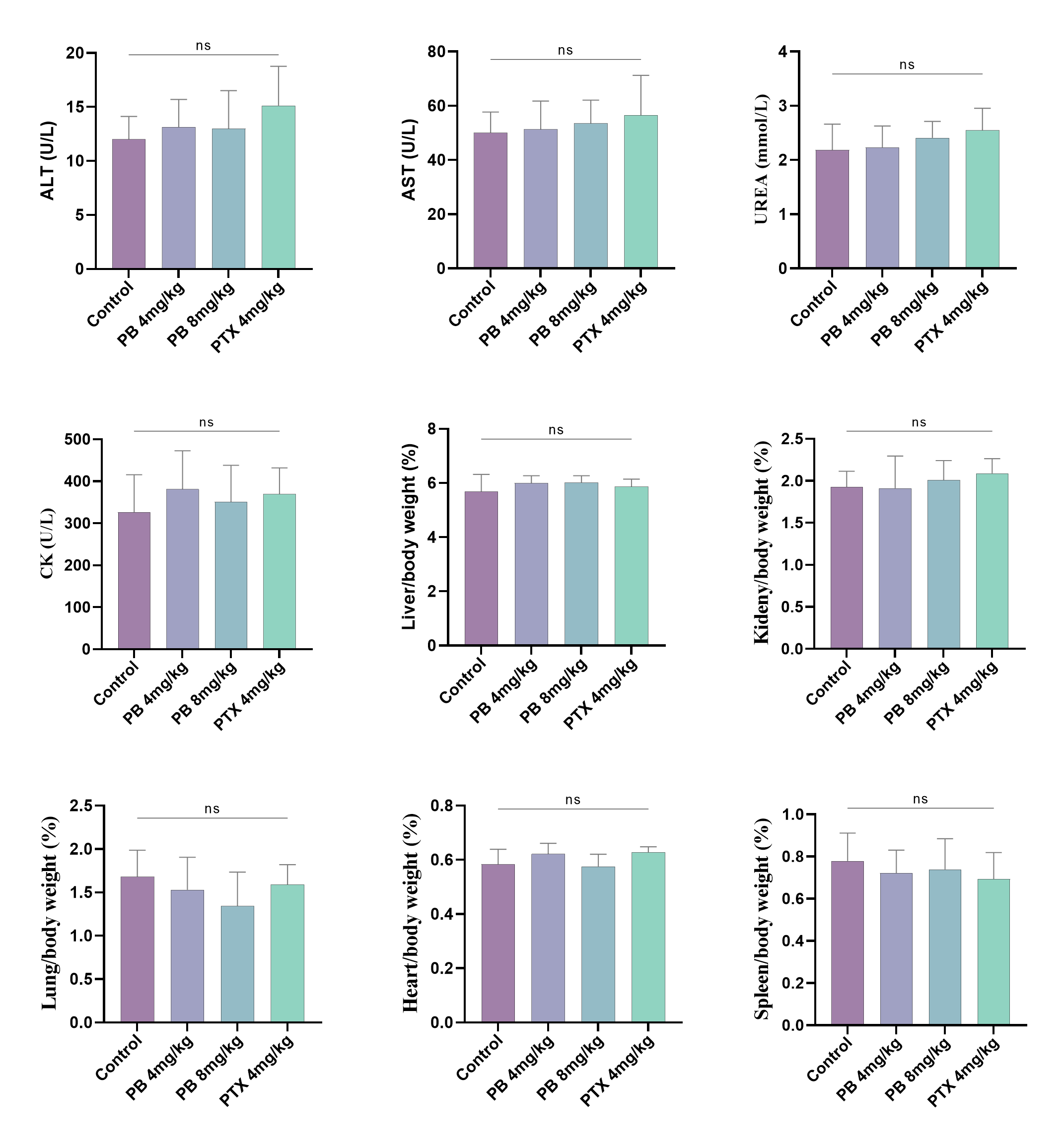
**Fig.S4. Biochemical indicators and organ indices in mice after PB treatment.

**Table S1: The top 100 compounds with the highest binding score to TRIB3**

| **No.** | **Name** | **CAS** | **Binding score** |
| --- | --- | --- | --- |
| 1 | Parishin B | 174972-79-3 | -9.529 |
| 2 | Ginsenoside Rg2 | 52286-74-5 | -9.227 |
| 3 | Catechin 7-O-β-D-glucopyranoside | 65597-47-9 | -9.226 |
| 4 | Polyphyllin I | 50773-41-6 | -9.225 |
| 5 | 11-Oxomogroside IIIE | 2096516-68-4 | -9.223 |
| 6 | Carubicin (hydrochloride) | 52794-97-5 | -9.223 |
| 7 | L-Valyl-L-phenylalanine | 3918-92-1 | -9.220 |
| 8 | Momordin IIc | 96990-19-1 | -9.220 |
| 9 | Rhamnetin | 90-19-7 | -9.217 |
| 10 | Aureusidin | 38216-54-5 | -9.215 |
| 11 | Crustacean cardioactive peptideree acid | 309247-84-5 | -9.215 |
| 12 | Gypenoside LI | 94987-10-7 | -9.215 |
| 13 | Episappanol | 111254-18-3 | -9.215 |
| 14 | Solasonine | 19121-58-5 | -9.214 |
| 15 | 9''-Methyl salvianolate B | 1167424-31-8 | -9.213 |
| 16 | (-)-Sparteine | 90-39-1 | -9.213 |
| 17 | Kukoamine B | 164991-67-7 | -9.206 |
| 18 | 4-(1,2-Dihydroxyethyl)benzene-1,2-diol | 28822-73-3 | -9.205 |
| 19 | Aloin(mixture of A&B) | 8015-61-0 | -9.204 |
| 20 | Kaempferol 3-neohesperidoside | 32602-81-6 | -9.204 |
| 21 | Isolugrandoside | 221660-27-1 | -9.203 |
| 22 | Methyldopa | 555-30-6 | -9.202 |
| 23 | Methyldopa (hydrate) | 41372-08-1 | -9.202 |
| 24 | Vindesine (sulfate) | 59917-39-4 | -9.200 |
| 25 | Isoliensinine | 6817-41-0 | -9.198 |
| 26 | Phlorizin | 60-81-1 | -9.197 |
| 27 | Salsolidine | 5784-74-7 | -9.194 |
| 28 | Mogroside II-?A | 1613527-65-3 | -9.192 |
| 29 | Celosin K | 1950579-53-9 | -9.189 |
| 30 | Quercetin-3-O-(6''-O-malonyl)-β-D-glucoside | 96862-01-0 | -9.189 |
| 31 | Lycoperodine-1 | 42438-90-4 | -9.188 |
| 32 | Moracin M | 56317-21-6 | -9.188 |
| 33 | 5-Hydroxymethyluracil | 4433-40-3 | -9.188 |
| 34 | Benzoyloxypaeoniflorin | 72896-40-3 | -9.188 |
| 35 | Diosmetin-7-O-β-D-glucopyranoside | 20126-59-4 | -9.188 |
| 36 | Isoacteoside | 61303-13-7 | -9.187 |
| 37 | Cyanidin 3-sambubioside (chloride) | 33012-73-6 | -9.184 |
| 38 | 11-Oxomogroside IIa | - | -9.184 |
| 39 | Aloin B | 28371-16-6 | -9.183 |
| 40 | Damulin B | 1202868-75-4 | -9.180 |
| 41 | Kushenol E | 99119-72-9 | -9.180 |
| 42 | Corilagin | 23094-69-1 | -9.178 |
| 43 | Canthin-6-one | 479-43-6 | -9.178 |
| 44 | GDP-L-fucose | 15839-70-0 | -9.176 |
| 45 | Sennoside B | 128-57-4 | -9.176 |
| 46 | N-(p-Coumaroyl) Serotonin | 68573-24-0 | -9.170 |
| 47 | S-Adenosyl-L-methionine (tosylate) | 52248-03-0 | -9.166 |
| 48 | Ligustroflavone | 260413-62-5 | -9.164 |
| 49 | Normetanephrine | 97-31-4 | -9.162 |
| 50 | Hyperoside | 482-36-0 | -9.160 |
| 51 | Tenuifoliside A | 139726-35-5 | -9.160 |
| 52 | Luteolin-3-O-beta-D-glucuronide | 53527-42-7 | -9.157 |
| 53 | Mangiferin | 4773-96-0 | -9.156 |
| 54 | Picfeltarraenin IA | 97230-47-2 | -9.155 |
| 55 | Genipin 1-β-D-gentiobioside | 29307-60-6 | -9.152 |
| 56 | Daphnetin | 486-35-1 | -9.152 |
| 57 | Kaempferol-7-O-β-D-glucopyranoside | 16290-07-6 | -9.151 |
| 58 | Garcinone C | 76996-27-5 | -9.151 |
| 59 | Apiopaeonoside | 100291-86-9 | -9.150 |
| 60 | Hydroxygenkwanin | 20243-59-8 | -9.149 |
| 61 | Dulcoside A | 64432-06-0 | -9.145 |
| 62 | Cyanidin-3-O-galactoside (chloride) | 27661-36-5 | -9.144 |
| 63 | Saikosaponin B4 | 58558-09-1 | -9.143 |
| 64 | Peonidin-3-O-galactoside (chloride) | 28148-89-2 | -9.143 |
| 65 | 2''-O-Rhamnosylicariside II | 135293-13-9 | -9.141 |
| 66 | Naringenin | 67604-48-2 | -9.141 |
| 67 | Imidazoleacetic acid (hydrochloride) | 3251-69-2 | -9.140 |
| 68 | 4-Hydroxymethylpyrazole | 25222-43-9 | -9.139 |
| 69 | UDP-3-O-acyl-GlcNAc (disodium) | **-** | -9.138 |
| 70 | Metanephrine | 5001-33-2 | -9.136 |
| 71 | Glycyrrhizic acid | 1405-86-3 | -9.134 |
| 72 | Dipotassium glycyrrhizinate | 68797-35-3 | -9.134 |
| 73 | UDP-glucosamine (disodium) | 1355005-51-4 | -9.130 |
| 74 | 2'-Deoxyguanosine | 961-07-9 | -9.129 |
| 75 | Beta-Zearalanol | 42422-68-4 | -9.129 |
| 76 | Ginsenoside F4 | 181225-33-2 | -9.129 |
| 77 | 1-Methylguanosine | 2140-65-0 | -9.128 |
| 78 | 12-Epinapelline | 110064-71-6 | -9.126 |
| 79 | 4-Methyldaphnetin | 2107-77-9 | -9.124 |
| 80 | Eriodictyol-7-O-glucoside | 38965-51-4 | -9.120 |
| 81 | Allantoic acid | 99-16-1 | -9.119 |
| 82 | Podophyllotoxin glucoside | 16481-54-2 | -9.118 |
| 83 | 1F-Fructofuranosylnystose | 59432-60-9 | -9.118 |
| 84 | Fraxetin | 574-84-5 | -9.116 |
| 85 | Methyl-β-D-Galactopyranoside | 1824-94-8 | -9.115 |
| 86 | 1-Methylinosine | 2140-73-0 | -9.113 |
| 87 | Spiramycin | 8025-81-8 | -9.109 |
| 88 | Isovitexin | 38953-85-4 | -9.108 |
| 89 | Flavanomarein | 577-38-8 | -9.107 |
| 90 | Harmalol (hydrochloride) | 6028-7-5 | -9.107 |
| 91 | 5,6-Dihydrouridine | 5627-5-4 | -9.107 |
| 92 | Naringenin | 480-41-1 | -9.106 |
| 93 | Fagomine | 53185-12-9 | -9.106 |
| 94 | Neolancerin | 117221-65-5 | -9.104 |
| 95 | Forsythoside B | 81525-13-5 | -9.104 |
| 96 | Epimedoside A | 39012-04-9 | -9.102 |
| 97 | 2'-Deoxyguanosine monohydrate | 312693-72-4 | -9.099 |
| 98 | trans-Zeatin | 1637-39-4 | -9.098 |
| 99 | Gossypetin | 489-35-0 | -9.098 |
| 100 | Demeclocycline (hydrochloride) | 64-73-3 | -9.098 |
